# History of genome editing in yeast

**DOI:** 10.1002/yea.3308

**Published:** 2018-02-26

**Authors:** Marcin G. Fraczek, Samina Naseeb, Daniela Delneri

**Affiliations:** ^1^ The University of Manchester, Faculty of Biology, Medicine and Health Manchester Institute of Biotechnology Manchester M1 7DN UK

**Keywords:** Cre‐*loxP*, CRISPR/Cas9, *delitto perfetto*, genome editing, yeast

## Abstract

For thousands of years humans have used the budding yeast Saccharomyces cerevisiae for the production of bread and alcohol; however, in the last 30–40 years our understanding of the yeast biology has dramatically increased, enabling us to modify its genome. Although S. cerevisiae has been the main focus of many research groups, other non‐conventional yeasts have also been studied and exploited for biotechnological purposes. Our experiments and knowledge have evolved from recombination to high‐throughput PCR‐based transformations to highly accurate CRISPR methods in order to alter yeast traits for either research or industrial purposes. Since the release of the genome sequence of S. cerevisiae in 1996, the precise and targeted genome editing has increased significantly. In this ‘Budding topic’ we discuss the significant developments of genome editing in yeast, mainly focusing on Cre‐loxP mediated recombination, delitto perfetto and CRISPR/Cas.

## 1 INTRODUCTION

The budding yeast Saccharomyces cerevisiae is one of the most extensively used model organism for studying eukaryotic functional genomics, metabolic pathways, aging, exploration of protein interactions and as a bio‐producer of a wide range of chemicals and by‐products (Sherman, 2002). The process robustness and the ease of maintenance and manipulation of this yeast make it a perfect candidate for the development of new genetically engineered strains for research and industrial applications. However, a number of other yeast species, termed ‘non‐conventional yeasts’ (van Dijken, 2002), have also attracted much attention and have been extensively used in research, as cell factories to produce recombinant proteins and biomolecules for various biotechnological, and for pharmaceutical purposes as well as to study pathogenicity. Examples of these are Candida albicans (Vyas, Barrasa, & Fink, 2015), *C. auris* (Defosse et al., 2017), C. glabrata (Ueno et al., 2007), Cryptococcus neoformans (Arras et al., 2016), *Hansenula polymorpha* (Saraya et al., 2012), *Kluyveromyces lactis* (Kooistra, Hooykaas, & Steensma, 2004), *K. marxianus* (Abdel‐Banat, Nonklang, Hoshida, & Akada, 2010), *Komagataella phaffii* (*Pichia pastoris*) (Weninger, Hatzl, Schmid, Vogl, & Glieder, 2016), *Scheffersomyces* (*Pichia*) *stipitis* (Maassen, Freese, Schruff, Passoth, & Klinner, 2008), *Schizosaccharomyces pombe* (Kim et al., 2010) and *Yarrowia lipolytica* (Kretzschmar et al., 2013). Genomic engineering in these species is, however, still more challenging compared with S. cerevisiae because of limited genomic and metabolic knowledge and/or less defined molecular tools. Better understanding of the biology of these organisms could not only significantly impact industrial production but also have relevance to human health.

Since the work on recombination in yeast in the early 1980s (Orr‐Weaver, Szostak, & Rothstein, 1981; Szostak, Orr‐Weaver, Rothstein, & Stahl, 1983), researchers all over the world have used molecular biology techniques to introduce recombinant DNA in order to alter S. cerevisiae traits. Genome sequencing of this yeast released to the public in 1996 opened exciting new opportunities to study S. cerevisiae (Goffeau et al., 1996). Many different systems have been developed for modification of chromosome sequence and structure within the cells. One of the most extensively used has been a PCR‐based gene targeting method (Wach, Brachat, Pohlmann, & Philippsen, 1994) that utilizes exogenous DNA introduced into the cell by various transformation methods (Kawai, Hashimoto, & Murata, 2010). Subsequently, the native double‐stranded break (DSB) repair system facilitates the manipulation of the yeast genome (sequence deletions, insertions, truncations, inversions, translocations or other types of mutagenesis). This method has been used to construct a comprehensive set of gene deletion mutants in S. cerevisiae (Giaever et al., 2002; Winzeler et al., 1999) and *S. pombe* (Kim et al., 2010). Recently, a non‐coding RNA deletion library has also been created in S. cerevisiae (Parker et al., 2017) by a similar approach.

A number of the gene engineering methods and techniques require the use of selectable markers for validation and maintenance of the integrated sequences. Both drug‐selectable markers and auxotrophic nutritional markers can be used in yeast, although the introduction of the latter in the genome can cause fitness changes, confounding the phenotypic effects of gene deletion and hampering the functional analysis (Baganz, Hayes, Marren, Gardner, & Oliver, 1997). The limited number of selectable markers makes it rather difficult to study the phenotypic effects of gene families, where multiple genes need to be deleted in the same background to reveal the phenotype (Delneri, Gardner, Bruschi, & Oliver, 1999). To overcome this problem, scientists have used a marker recycling method, exploiting site‐specific recombinase technologies that utilize recombinases. This is particularly useful if retention of the selectable marker in the genome is not desirable or the same selectable marker is to be used to delete another gene (marker recycling). Cre‐*loxP‐*mediated recombinase and *delitto perfetto* are good examples of such a system.

However, in the last few years, the introduction of the CRISPR system has revolutionized the area of precision genome engineering in many organisms, including yeast.

In this mini‐review we describe the most important methods applied for genome editing in yeast, mostly focusing on Cre‐*loxP‐*mediated recombination, *delitto perfetto* and CRISPR/Cas9.

## 2 DOUBLE‐STRANDED BREAKS REPAIR MECHANISM

**Figure 1:**
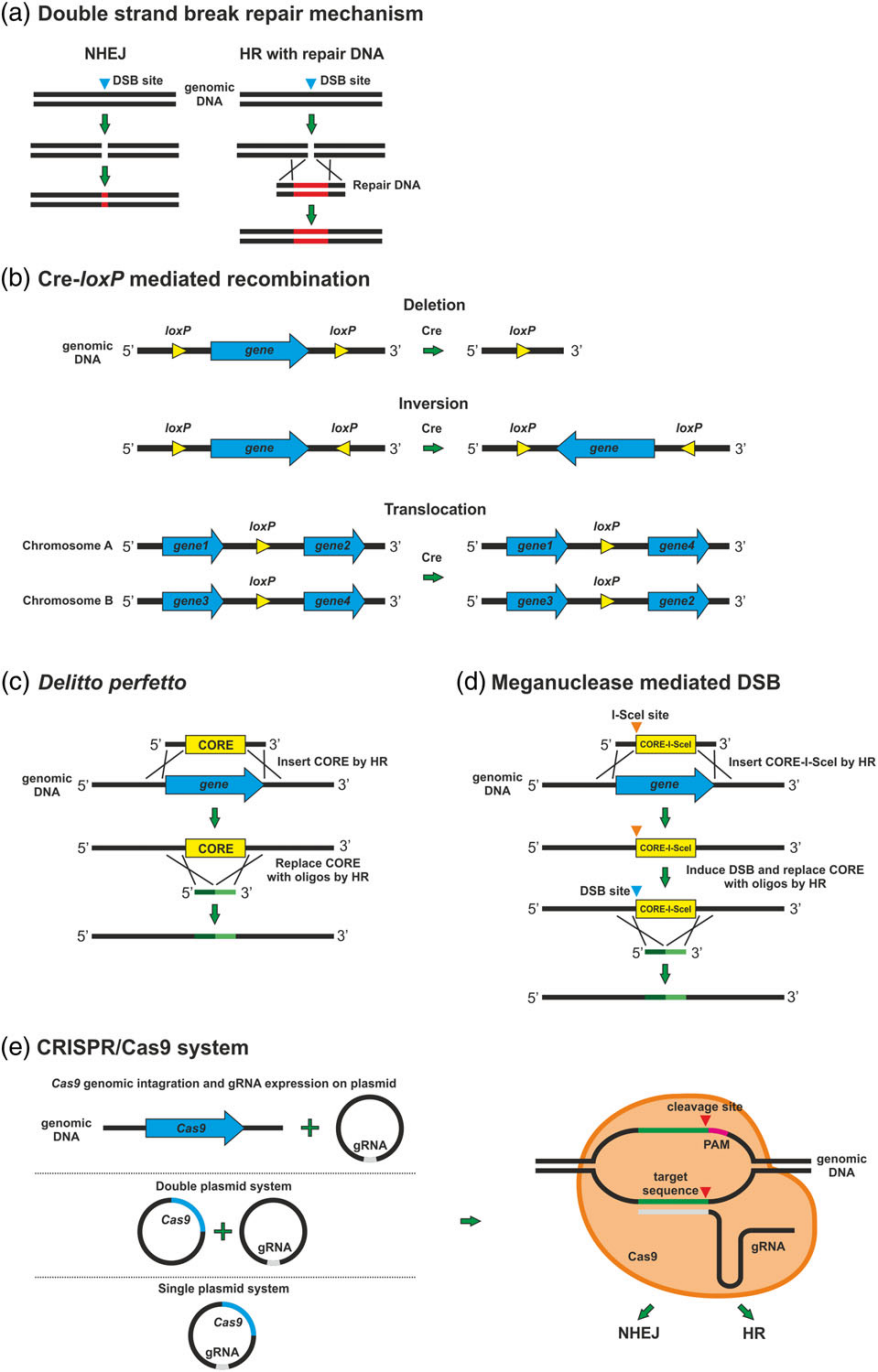
Overview of the yeast genome editing methods described here. (a) Double strand break (DSB) mechanism mediated either by non‐homologous end joining (NHEJ) or homologous recombination (HR). A repair DNA is used to increase the efficiency of genome editing by HR. (b) Cre‐*loxP* mediated recombination. Depending on the orientation and location of the *loxP* sequences, activated Cre recombinase can catalyse deletions, inversions or translocation of a chromosomal fragment. (c) In *delitto perfetto* a CORE cassette is used to replace a gene or sequence of interest by HR. Subsequently, the CORE cassette is removed by HR using oligonucleotides complementary to the flanking regions of the cassette. (d) Similarly to *delitto perfetto*, the meganuclease mediated DSB method utilizes a CORE cassette, however the addition of an I‐SceI site and induction of DSB significantly increase the recombination rate. (e) CRISPR/Cas9. A single or double plasmid system are used to express the Cas9 endonuclease and guide RNA (s) (gRNA). Alternatively, the *Cas9* gene is integrated into the yeast genome and the gRNA is delivered on a plasmid. After expression, the gRNA locates the target sequence and the endonuclease cleaves the foreign DNA that subsequently leads to either NHEJ or HR events [Colour figure can be viewed at http://wileyonlinelibrary.com]

A crucial step for genome editing is creation of DSBs at the locus to be modified and their repair. The repair is achieved by two major pathways in yeast (Figure 1a): (a) homologous recombination (HR), which depends on sequence homology; and (b) non‐homologous end joining (NHEJ), which is more error prone and involves integration between regions of little or no homology (Clikeman, Khalsa, Barton, & Nickoloff, 2001). The HR pathway is very efficient in S. cerevisiae (Lorenz et al., 1995) and plays a dominant role in DSB repair, requiring only 38–50 bp of target gene homology of both sides of the marker cassette (Baudin, Ozier‐Kalogeropoulos, Denouel, Lacroute, & Cullin, 1993; Sonoda, Hochegger, Saberi, Taniguchi, & Takeda, 2006). The NHEJ pathway in S. cerevisiae is mainly observed if the HR mechanism is blocked and depends on the ku complex proteins yKu70p and yKu80p (*HDF1* and *HDF2*, respectively; Clikeman et al., 2001; O'Driscoll & Jeggo, 2006). Deletion of *HDF1* and *HDF2* in *S. cerevisiae* leads to defects in recombination and temperature sensitivity, and does not improve already very efficient HR events (Cervelli & Galli, 2000; Clikeman et al., 2001; Mathiasen & Lisby, 2014).

Most other yeast species favour the NHEJ pathway over HR, even when exogenous DNA is introduced, making precise gene editing difficult and inefficient (Dmytruk, Voronovsky, & Sibirny, 2006; Kegel, Martinez, Carter, & Astrom, 2006; Kooistra et al., 2004; van Dijk et al., 2001). Moreover, large sequences of target gene flank homology do not guarantee efficient mutagenesis, which results in laborious and time‐consuming phenotypic and PCR‐based screening to select desirable mutants. Therefore, genome engineering in non‐conventional yeasts has mostly been obtained by random integration that sometimes leads to unexpected and unwanted genomic and phenotypic changes. As shown in other fungi (Choquer et al., 2008; Collopy et al., 2010; da Silva Ferreira et al., 2006), enhancing HR or eliminating NHEJ could be the way forward to improve homologous integrations and could increase the editing efficiency, and has also been introduced in yeast (Kooistra et al., 2004; Maassen et al., 2008; Ueno et al., 2007). For example, Kooistra et al. (2004) have demonstrated that disruption of the *KU80* gene in *K*. *lactis* increased the HR events to 97%. Similarly, *KU70* or/and *KU80* disruption has improved the targeted gene integration in *S. stipitis* (Maassen et al., 2008), *Y. lipolytica* (Kretzschmar et al., 2013), *H. polymorpha* (Saraya et al., 2012), *K. phaffii* (Naatsaari et al., 2012), C. glabrata (Ueno et al., 2007) and *K. marxianus* (Abdel‐Banat et al., 2010).

## 3 CRE‐*LOXP* MEDIATED RECOMBINATION

Cre‐mediated recombination is a powerful tool to generate genomic rearrangements and overexpression of genes. It was developed by Sauer (1987) in S. cerevisiae and later modified and improved by other groups (Carter & Delneri, 2010; Delneri et al., 2000; Guldener, Heck, Fielder, Beinhauer, & Hegemann, 1996). This system has also been applied in non‐*Saccharomyces* yeasts, such as *S. pombe* (Avelar, Perfeito, Gordo, & Ferreira, 2013; Hentges, Van Driessche, Tafforeau, Vandenhaute, & Carr, 2005; Iwaki & Takegawa, 2004), *K. lactis* (Steensma & Ter Linde, 2001), *K. marxianus* (Ribeiro, Gombert, Teixeira, & Domingues, 2007), *Y. lipolytica* (Fickers, Le Dall, Gaillardin, Thonart, & Nicaud, 2003), *K. phaffii* (Marx, Mattanovich, & Sauer, 2008 *)*, C. albicans (Dennison, Ramsdale, Manson, & Brown, 2005) and *H. polymorpha* (Krappmann, Pries, Gellissen, Hiller, & Braus, 2000). Moreover, it has been successfully used in other model systems such as in mouse (Gu, Marth, Orban, Mossmann, & Rajewsky, 1994), *Drosophila* (Siegal & Hartl, 1996), *Xenopus* (Werdien, Peiler, & Ryffel, 2001), zebrafish (Dong & Stuart, 2004) and plants (Gilbertson, 2003). This method involves the use of marker cassette flanked by two direct repeats of 34 bp small recognition sequences, called *lox*, *loxP*, *lox2272* and *loxLE*/*RE* (Figure 1b; Carter & Delneri, 2010). This cassette not only possesses a dominant heterologous antibiotic resistance marker that replaces the gene of interest, but it also facilitates the excision of the markers from the genome by Cre‐mediated recombination between the two flanking *loxP* sites. Therefore, by the action of the Cre recombinase, the selectable marker can be rescued, leaving only one *loxP* ‘scar’ in the genome. The key benefit of this system is that the Cre recombinase can catalyse either multiple gene deletions (Akada et al., 2006), inversions (Naseeb et al., 2016; Naseeb & Delneri, 2012) or translocations of a chromosomal fragment (Delneri et al., 2003) depending on the orientation and location of *loxP* sequences flanking that fragment. When multiple deletions is the objective of the study, the availability of multiple different *loxP* sequences (Carter & Delneri, 2010) can prevent chromosomal rearrangements. In fact, Cre recombinase can recognize identical *lox* sites (i.e. *loxP* and *loxP*; *lox2272* and *lox2272*) but cannot recombine between different *lox* scars (i.e. *loxP* and *lox2272*), preventing unwanted rearrangements.

Chromosomal rearrangements such as translocations and inversions are known to affect the mitotic and meiotic fitness of different yeast species (Avelar et al., 2013; Colson, Delneri, & Oliver, 2004; Naseeb et al., 2016; Naseeb & Delneri, 2012). It has been shown that S. cerevisiae strains carrying single gene inversions can cause fitness defects in nutrient‐limited media (Naseeb & Delneri, 2012). However, large chromosomal inversions in S. cerevisiae can be either lethal or neutral depending upon the environmental conditions (Naseeb et al., 2016). Similar findings were observed by Avelar et al. (2013) in *S. pombe*, showing that inversions and translocations can have beneficial or deleterious growth affects for both meiotic and mitotic fitness. Chromosomal inversions can also cause transcriptional alterations in the genome of yeast species.

More recently, the Cre‐*loxP* system has been used to produce S. cerevisiae synthetic chromosomes (synthetic yeast Sc2.0 project) with all non‐essential genes flanked by *loxPsym* sites. Subsequently, Cre is triggered to rearrange the genome (Annaluru et al., 2014; Dymond et al., 2011; Shen et al., 2016). This process, referred to as SCRaMbLEing, enables genome minimization, which in turn will provide insight into yeast evolution, properties of chromosomes, genome organization, RNA splicing, etc. Synthetic yeast may also be important for industrial purposes.

## 4 *DELITTO PERFETTO* AND MEGANUCLEASES

*Delitto perfetto* has been widely used for genome alterations via HR in S. cerevisiae (Storici, Lewis, & Resnick, 2001). This method combines different synthetic oligonucleotides for targeting the gene of interest with the practicality of a general selection system. The application of various counter selectable markers and reporter genes (CORE) allow for efficient genome editing (Figure 1c). One of the main features of this technique is the ability to eliminate any marker sequences used for selection, ensuring no foreign DNA is left in the genome, which may cause unforeseen effects. Compared with the other methods, *delitto perfetto* is simple and can easily be used for any kind of genetic modification, from a single or multiple nucleotide mutation to large deletions or chromosomal translocations (Langle‐Rouault & Jacobs, 1995; Scherer & Davis, 1979).

To further increase the transformation efficiency by HR, break‐mediated methods have attracted much attention (Storici, Durham, Gordenin, & Resnick, 2003; Storici & Resnick, 2003). The use of homing endonucleases or meganucleases such as mitochondrial I‐SceI to induce a single DSB at the locus to be modified and stimulate oligonucleotide targeting greatly increases recombination of homology sequences and integration efficiency, even of large DNA constructs (Figure 1d). One of the drawbacks of this method is that mutagenesis is restricted to the genomic region surrounding the inserted cassette and it cannot be applied to the applications where selectable markers are required.

## 5 CRISPR/CAS9

In the past few years, CRISPRs (clustered regularly interspaced short palindromic repeats) has revolutionized the field of genome engineering owing to their simplicity and high efficacy. It enables fast and reliable genetic manipulation in organisms it has been used in and it only requires two components to work: a guide RNA (gRNA), e.g. under an RNA polymerase III promoter, and the nuclear localization tag fused DNA endonuclease, with Cas9 being the most commonly used. The great advantage of this system is the generation of DSBs in desired locus or loci by the action of the endonuclease and single or multiple gene editing achieved by cell native repair system.

CRISPRs were first discovered by Ishino, Shinagawa, Makino, Amemura, and Nakata (1987) in Escherichia coli as nucleotide repeats interspaced with short DNA sequences. Several years later, it was shown that the CRISPR sequences and associated with them Cas proteins serve as a bacterial and archeal anti‐viral defence mechanism (Barrangou et al., 2007; Bolotin, Quinquis, Sorokin, & Ehrlich, 2005; Mojica, Diez‐Villasenor, Garcia‐Martinez, & Soria, 2005; Pourcel, Salvignol, & Vergnaud, 2005). *Streptococcus pyogenes* CRISPR/Cas immune mechanism is currently one of the best characterized (Jinek et al., 2012) and the Cas9 originating from *S. pyogenes* is the most commonly used in yeast research, although other endonucleases such as Cpf1 (Cas12a) also arouse scientists’ interest. In the naturally occurring system, Cas proteins incorporate foreign DNA (protospacer) into a CRISPR array in form of a spacer between identical native palindromic repeats. Subsequently, the CRISPR array is transcribed to CRISPR RNA (crRNA) protospacers and the crRNAs hybridize to *trans*‐acting RNAs (tracrRNAs), creating a crRNA–tracrRNA hybrid. This hybrid associates with the type‐II endonuclease Cas9 and the whole complex recognizes and cleaves the foreign DNA that is complementary to the protospacer, creating a DSB. A 20 bp target complementary sequence within crRNA is required for Cas9 activity, localized immediately upstream of the protospacer adjustment motif (PAM) which is composed of three nucleotides NGG required for cutting. Any genomic loci followed by the 5′‐NGG‐3′ PAM sequence can be targeted for modification. The engineered CRISPR/Cas9 system utilizes the guide RNA (gRNA) composed of fused sequences of crRNA and tracrRNA. By modifying the 20 bp of the 5′ end of the gRNA molecule, the system can be used to target any desired sequence in the genome for editing (Figure 1e; Jinek et al., 2012). Upon DNA cleavage, the cell must repair the DNA break in order to survive. Introduction of desired sequence modifications is then achieved by exogenously supplied donor DNA (repair DNA).

Efficient gene engineering is not the only advantage of the CRISPR system. It has also been demonstrated to have capacity for activation and repression (interference) of gene transcription in yeast, which has been reported in several studies (Deaner & Alper, 2017; Farzadfard, Perli, & Lu, 2013; Gilbert et al., 2013; Lenstra, Coulon, Chow, & Larson, 2015), but this is beyond the focus of this review.

Over the last few years, several different approaches have been reported to delete genes or integrate DNA sequences into genome in various yeast backgrounds (Horwitz et al., 2015; Jacobs, Ciccaglione, Tournier, & Zaratiegui, 2014; Schwartz, Hussain, Blenner, & Wheeldon, 2016; Vyas et al., 2015). They usually involve single or double plasmid systems carrying gRNA and the *Cas9* gene, and/or a donor DNA targeting the desired sequence. Some studies have also reported stable integration of the *Cas9* gene into the genome, while the gRNA was expressed from a plasmid (Mans et al., 2015). DiCarlo et al. (2013) were the first to report a single gene disruption in a yeast (S. cerevisiae) haploid strain by HR using the CRISPR/Cas9 system. They demonstrated that targeted DSBs can increase HR in a constitutively expressing Cas9 strain by 130‐fold when transformed with a double‐stranded 90 bp oligonucleotide containing the homologous ends to the target sequence and a transient gRNA cassette. Their double plasmid system approach resulted in near 100% recombination of the donor DNA and a single gene disruption.

A double plasmid system was also used for example by Bao et al. (2015) in S. cerevisiae to delete *CAN1* and *ADE2* genes, in which Cas9 and tracrRNA are expressed on one plasmid and CRISPR array on another. The efficiency was only 14.7% and 12.5% for *CAN1* and *ADE2*, respectively. Changing the strategy to a single plasmid system improved the knock‐out efficiency to 100% for both genes. On the other hand, a two‐plasmid system can provide an engineering efficiency of 85–100% in a haploid strain by simultaneously introducing three exogenous genes into separate loci (Ronda et al., 2015).

A similar strategy to the one reported by DiCarlo et al. (2013), using a Cas9 expressing haploid strain and a 90 bp donor DNA, was used by Jakočiūnas et al. (2015) to engineer a promoter region of a gene and delete four other genes in S. cerevisiae involved in the production of mevalonate, a key precursor for industrially important isoprenoid production. High transformation efficiency was reported, although no obvious phenotype for single knock‐out mutants was observed. They also generated plasmids with one to five gRNAs (single plasmid strategy) to simultaneously knock out genes with efficiency of 50–100% (multiplex CRISPR).

Ryan et al. (2014) successfully deleted 11 unlinked yeast genes in a diploid background of S. cerevisiae using 120 bp oligonucleotides with 100% efficiency. Moreover, the authors generated a plasmid (single plasmid system) carrying the *Cas9* gene and three different gRNA expression cassettes, and performed triple gene deletions using a multiplex CRISPR approach. The efficiency was only 19% in the diploid strain but near 100% in the haploid. Using a similar methodology to that for the gene knock‐out, they assembled *in vivo* three overlapping DNA fragments carrying nourseothricin‐resistance marker and integrated them into the *URA3* locus. The efficiency was 85% in a diploid strain and 70% in a polyploid strain. Lower efficiency of 15–60% by knocking out four genes one by one (*URA3*, *HIS3*, *LEU2* and *TRP1*) in an industrial polyploid strain was reported by Zhang et al. (2014).

A single plasmid carrying the *Cas9* gene and multiple gRNAs was also used by Generoso, Gottardi, Oreb, and Boles (2016) in S. cerevisiae, which enabled simultaneous deletion of multiple genes in haploid and diploid backgrounds. Plasmids were either constructed *in vitro* or assembled *in vivo* using yeast HR systems. Around 90% deletion efficiency was reported using the *in vitro* generated plasmids for one to three loci deleted at once in the haploid backgrounds and one to two loci in the diploid background. However, the transformation efficiency for the *in vivo* assembled plasmid in both backgrounds was from 0 to 60%.

CRISPR/Cas9 has also been extended to other yeast species. For instance, in *K. lactis*, Horwitz et al. (2015) engineered a muconic acid pathway (composed of six genes) by simultaneously generating three DSBs for HR‐mediated gene integration. To this end, the *Cas9* gene was integrated at the *GAL80* locus and the *KU80* gene was deleted to minimize the effect of NHEJ. The expression of gRNAs was driven by the *SNR52* polymerase III promoter and the gRNAs were delivered into the cells on single plasmids or transformed as linear PCR fragments along with backbone linear vectors and reconstructed into circular plasmids *in vivo* (gap repair). The donor DNAs were also delivered into the cells as linear PCR products. Although the rate of triple integration was only 2.1%, the authors were able to demonstrate that the application of CRISPR/Cas9 system could greatly decrease the time needed to engineer metabolic pathways in this yeast.

The genome of *Y. lipolytica*, a yeast used in the industry to produce citric acid, intracellular lipids and lipase (Goncalves, Colen, & Takahashi, 2014), has also been modified using the CRISPR/Cas9 system. Schwartz et al. (2016) constructed a codon‐optimized Cas9 *Y. lipolytica* strain and tested various promoters to express the gRNA on a centromeric plasmid. The highest disruption efficiencies for their NHEJ system for gene *PEX10* were achieved with a *SCR1*–tRNA promoter (>90% after 4 days of outgrowth) and a *SNR52*−tRNA promoter (>80% after 4 days of outgrowth). Subsequently, they tested the efficiency of the HR system by deleting the *KU70* gene and targeting another (*PEX10*). The efficiency was 86% in this case.

Another group (Gao et al., 2016) created a *Y. lipolytica* codon‐optimized Cas9 and used a single plasmid system to deliver the gRNA in order to test the efficiency of NHEJ and HR. After 4 days of outgrowth, 86, 37 and 19% efficiency was achieved for one, two or three targeted genes, respectively, when no donor DNA was present. A higher rate of efficiency occurred when the donor DNA was delivered in the *KU70/80* mutants.

Efforts have been made to enhance the genome engineering capabilities in the important recombinant producer *K. phaffii*. In the study by Weninger et al. (2016) the authors tested various codon‐optimized *Cas9* genes and *Cas9* promoters, and different gRNA sequences as well as gRNA promoters; however, only ~6% of their tested constructs were functional for efficient genome editing. The use of human codon‐optimized Cas9 and the ribozyme‐flanked gRNA resulted in the highest NHEJ efficiency (~90% for a single gene nonsense mutation and ~70% for two genes). Introducing the donor DNA containing ~1 kb gene homologous sequences into the cell provided a very low integration efficiency of ~2.5%. This suggests that, although there is a potential to engineer *K. phaffii* genome by donor DNA, the preferred DSB repair system remains NHEJ.

The CRISPR/Cas9 system has also been used to engineer the genome of pathogenic yeasts such as C. albicans (Vyas et al., 2015), C. glabrata (Enkler, Richer, Marchand, Ferrandon, & Jossinet, 2016) and C. neoformans in order to study the gene functions in virulence. Genome molecular manipulation is especially difficult in these yeasts owing to their complex biology and lack of specialized tools. In C. albicans, the *Cas9* gene was codon optimized for the expression in all CTG clade species, integrated into the genome and expressed under the *ENO1* promoter. The gRNA was expressed under the *SNR52* RNA polymerase III promoter. The deletion efficiency of the *ADE2* gene ranged between 20 and 80% depending on the approach.

In another study, two centromeric plasmids with *Cas9* and gRNA sequences were used to manipulate the genome of C. glabrata (Enkler et al., 2016). For the gRNA expression, the authors used the S. cerevisiae *SNR52* promoter as well as the promoter of C. glabrata *RNAH1* followed by the tRNA *Tyr 2* terminator. The latter was shown to be more efficient in this yeast to generate indels in the *ADE2* gene by NHEJ. Introduction of donor DNA further increased the transformation efficiency.

In C. neoformans, the expression of Cas9 was achieved by either integrating the *Cas9* gene into the genome (Arras et al., 2016) or delivering it on a linear vector (Wang et al., 2016). In both cases, the gRNA was introduced into the cells on a linear vector. Wang et al. (2016) showed that the efficiency of this system to generate nonsense mutations without the donor DNA was up to 80% and with the donor DNA ranged from 20 to 90%. Arras et al. (2016) showed that the HR events occur in this fungus with ~70% efficiency using the CRISPR/Cas9 system.

Although the CRISPR/Cas9 system provides several advantages compared with other techniques, it also has some limitations. First of all, it requires the PAM motif (usually ‐NGG‐) to be recognized by the Cas9 nuclease and the DSB only occurs downstream of this sequence (Jinek et al., 2012). Therefore, the CRISPR/Cas9 system can only be applied to sequences with proximal to the PAM motifs. A solution is a discovery or engineering of new Cas nucleases that require motifs other than ‐NGG‐ PAM (Mitsunobu, Teramoto, Nishida, & Kondo, 2017; Schwartz et al., 2016). Another disadvantage of the CRISPR/Cas9 system is an off‐site effect, which occurs when the nucleotides that drive the CRISPR/Cas9 complex recognize target sequences with mismatched bases, generating undesirable multiple DSBs (Hsu et al., 2013). The off‐side effects can be difficult to recognize and usually require scanning of the entire genome. However, in an organism with HR as the preferred repair mechanism, the off‐site effects would be expected to be less profound. In the study by Ryan et al. (2014) several off‐site mutations were found in the CRISPR/Cas9 edited yeast strains but the authors concluded that these were unlikely to occur owing to the activity of Cas9.

## 6 CONCLUSION

The last three decades have witnessed a great development in the field of yeast genome engineering. Since the pioneering work in the 1980s on recombination, several new methods and techniques have been introduced. This has been particularly important for yeast strains used for industrial purposes for which an increase in the production of certain metabolites or elimination of others was desired but also for researchers who use S. cerevisiae as a model organism.

For several years, gene knock‐out has been carried out by introducing a linear double‐stranded deletion cassette containing a marker flanked with complementary regions to the target locus. Although this method is still widely used, new methods of precise genome engineering continue to emerge. Recently, the development of the CRISPR/Cas9 system has revolutionized the field of molecular biology, not only in yeast but also in a range of other organisms. By generating DSBs and thus stimulating HR, it provides a highly reliable genome engineering tool that enables one‐step and marker‐free single or multiple gene deletion or integration. The rapid pace of development and improvement of genome editing tools makes it an exciting and important leap in the era of biotechnology.
